# Preoperative Oral Rehydration Therapy Mitigates Circulatory Suppression During Anesthesia Induction: A Retrospective Study of 1,000 Elective Surgery Cases Exploring Appropriate Preoperative Intake Volume

**DOI:** 10.7759/cureus.102945

**Published:** 2026-02-04

**Authors:** Hideki Taniguchi, Takaaki Kamada, Toshio Sasaki, Motokazu Koga

**Affiliations:** 1 Patient Support Center, Saiseikai Yokohamashi Tobu Hospital, Yokohama, JPN; 2 Anesthesiology, Kanagawa Cancer Center, Yokohama, JPN; 3 Perioperative Support Center, Saiseikai Yokohamashi Tobu Hospital, Yokohama, JPN

**Keywords:** oral rehydration solution, preoperative intake volume, preoperative oral rehydration therapy, preoperative volume control, prevention of circulatory suppression

## Abstract

Background and objective

Preoperative oral rehydration therapy has been introduced as an alternative to conventional IV fluid management. It helps shorten the preoperative fasting period, prevents and corrects dehydration before surgery, and supports circulatory stability during anesthesia induction. Appropriate preoperative oral rehydration solution (ORS) intake may help alleviate circulatory fluctuations. This study aimed to examine the relationship between ORS intake volume and circulatory fluctuations during anesthesia induction in 1,000 elective surgery cases.

Methods

We conducted a retrospective study at an independent administrative institution affiliated with the Kanagawa Prefectural Hospital Organization. We examined 1,000 patients who underwent elective surgery under general anesthesia between April 1, 2021, and December 27, 2021. Eligible patients were provided with three 500 mL bottles of ORS within 12-18 hours before surgery. Patients were classified into two groups: those who received vasopressors after anesthesia induction (V group) and those who did not (N group). Demographic and clinical data were obtained from electronic medical and anesthesia record systems. We examined whether differences in patient demographics and anesthesia-related factors influenced vasopressor administration. The primary outcome was the dose-response relationship between ORS intake volume and vasopressor administration, while secondary outcomes included identifying patient- and anesthesia-related factors influencing vasopressor use.

Results

Of the 1,000 patients enrolled, 473 (47%) received vasopressors between anesthesia induction and the start of surgery. ORS intake of <800 mL increased the rate of vasopressor administration from 50% to 52%. In contrast, ORS intake of ≥ 800 mL reduced the rate of vasopressor administration, reaching a minimum of 40%. Multivariable analysis revealed that ORS intake of ≥ 800 mL was significantly associated with a lower frequency of vasopressor administration.

Conclusions

Appropriate preoperative ORS intake may help mitigate circulatory suppression during anesthesia induction. Analysis of 1,000 patients undergoing elective surgery revealed an association between an ORS intake volume of 800-1,500 mL and reduced vasopressor requirements during anesthesia induction. The risk of hypotensive events was particularly high in patients receiving total IV anesthesia with combined epidural analgesia, older patients, underweight patients, those who skipped dinner the night before surgery, and patients with higher American Society of Anesthesiologists Physical Status classification.

## Introduction

A 2009 study introduced the concept of preoperative oral rehydration therapy (POORT) as an alternative method of fluid management to conventional IV fluid therapy [1]. POORT helps shorten the preoperative fasting period, prevents and corrects dehydration before surgery, and ensures safety during anesthesia induction [2]. Our single- and multicenter collaborative studies demonstrated that POORT provides an effect equivalent to that of preoperative IV fluid therapy in terms of fluid and electrolyte replenishment. It is a safe method of preoperative fluid management that does not increase gastric fluid volume before general anesthesia induction. Additionally, it can alleviate preoperative thirst, hunger, and discomfort associated with IV fluid administration [1,3].

Based on these findings, the Japanese Society of Anesthesiologists published preoperative fasting guidelines in July 2012, permitting the intake of clear fluids up to two hours before anesthesia induction, regardless of age [4]. However, POORT is not expected to have a similar effect as preoperative intake of a hyper-concentrated carbohydrate drink (HCHO), which is recommended in the Enhanced Recovery After Surgery guidelines and involves preoperative intake of HCHO (HCHO loading) containing 12.6% glucose to suppress postoperative insulin resistance [5]. For HCHO loading, the carbohydrate load target is set at 100 g (intake volume = 800 mL) the night before surgery and 50 g (intake volume = 400 mL) on the day of surgery. In contrast, POORT intake is generally based on an amount equivalent to that of the IV fluids previously administered before surgery, and there is no established evidence regarding a specific preoperative intake volume.

In one study, fluid volume before anesthesia induction was assessed using bioimpedance analysis, and it was found that the oral rehydration solution (ORS) intake required to achieve a preoperative zero balance was 21.9 ± 2.3 mL/kg. However, this was a small-scale study focusing on patients undergoing gastrectomy, and the hemodynamic effects during anesthesia induction were not evaluated [6]. The goal of POORT is to maintain preoperative fluid volume and mitigate circulatory suppression during anesthesia induction [1,2]. A previous systematic review reported that intraoperative hypotension lasting more than 10 minutes can lead to organ dysfunction in patients undergoing noncardiac surgery [7]. Furthermore, a previous study on elective cesarean sections using combined spinal-epidural anesthesia evaluated the safety of POORT during anesthesia induction based on the required dose of vasopressors [8].

In the present study, we hypothesized that an appropriate preoperative intake of ORS reduces circulatory suppression from anesthesia induction to the start of surgery. To test this hypothesis, we retrospectively examined the relationship between ORS intake volume and circulatory fluctuations during anesthesia induction in 1,000 cases. The findings of this study are expected to improve the safety of anesthesia management by clarifying the association between preoperative ORS intake volume and hemodynamic stability.

Our study center, the Kanagawa Cancer Center, an independent administrative institution under the Kanagawa Prefectural Hospital Organization, was selected because of its standardized protocol for POORT implementation, where the only preoperatively permitted fluid intake was ORS. Consequently, preoperative fluid management at our center was conducted exclusively through oral rehydration therapy, enabling us to assess the effects of ORS intake on anesthesia induction.

## Materials and methods

Study design, patient selection, and perioperative management

This study was designed as a retrospective review of electronic medical records. Following the eligibility criteria, we sequentially enrolled patients who underwent elective surgery under general anesthesia at our center from April 2021 onward. Registration was completed once 1,000 cases were included, which constituted the study cohort. Eligibility criteria were (1) age ≥20 years with no upper limit; (2) American Society of Anesthesiologists Physical Status (ASA PS) classification I or II; and (3) indication for POORT. The exclusion criterion was ORS intake of <500 mL. Table 1 shows the criteria for POORT implementation at our center, and Table 2 details the POORT procedure.

**Table 1:** Criteria for POORT at our center ASA PS, American Society of Anesthesiologists Physical Status; POORT, preoperative oral rehydration therapy

Category	Criteria
Indication	Patients scheduled for surgery who have consented to POORT will not be given presurgical medications (sedatives, painkillers, etc.)
Relative contraindication	These conditions are considered high-risk and apply only to patients approved by the anesthetist:
	1. History of surgical procedures involving the upper digestive tract (stomach, esophagus, etc.) or the liver, gallbladder, or pancreas
	2. Use of medications that suppress digestive tract peristalsis
	3. Severe obesity expected to complicate mask ventilation
	4. Anticipated severe difficulty with intubation
	5. Dysphagia or recurrent laryngeal nerve paralysis (unable to eat normal food)
	6. Symptoms of increased intracranial pressure
	7. Cognitive impairment preventing the patient from understanding how to drink fluids
	8. Treatment for diabetes with a history of diabetic coma
Absolute contraindication	1. Refusal of POORT or request for IV rehydration by the patient
	2. Physician judgment that POORT is inappropriate due to the surgical procedure or anesthesia management
	3. Inability of the patient to eat or drink due to dysphagia, stenosis, ileus, or similar conditions
	4. Risk classified as ASA PS III or higher

**Table 2:** Implementation of POORT at our center ORS, oral rehydration solution; POORT, preoperative oral rehydration therapy

Category	Instructions
Amount and method of drinking ORS	1. Drink 500-1,500 mL of ORS, as much as you are able to consume.
	2. ORS is absorbed quickly. If a large amount is consumed at once, it will be excreted through urine, sweat, and other body fluids. Therefore, drink small amounts at a time.
	3. ORS can be consumed at room temperature or chilled after refrigeration.
	4. Eating and drinking water or other liquids besides ORS are prohibited.
Last drinking time	1. For surgeries starting at 9:00 AM, the anesthesiologist will give instructions up to 6:00 AM on the day of surgery; for other surgery times, instructions are given up to three hours before the scheduled entry to the operating room.
	2. If the entry time to the operating room changes on the day of surgery, the anesthesiologist may adjust the last drinking time to at least two hours before entry.
If the consumed volume is <500 mL	1. For morning admissions, no special consideration is required.
	2. For admissions after 1:00 PM, if ORS intake is less than 500 mL, infusion therapy will be administered.
Time of distribution of ORS	After dinner on the night before surgery, patients will be provided with 1,500 mL of ORS.

At our center, the ORS used for POORT was OS-1^®^ (Otsuka Pharmaceutical Factory, Tokushima, Japan), and its composition is presented in Table 3. Eligible patients were provided with three 500 mL bottles of ORS within 12-18 hours before surgery (Table 2) and were allowed to consume 500-1,500 mL at their own pace, with all other fluid and food intake prohibited. Dinner was permitted the night before surgery, and a laxative was administered depending on the surgical procedure. 

**Table 3:** Composition of ORS used in this study ORS, oral rehydration solution

Item	Composition	Unit	Numerical value
Volume	-	mL	500
Energy	-	kcal	50
Ingredients	Na⁺	mEq/L	50
	K⁺	mEq/L	20
	Cl⁻	mEq/L	50
	Glucose	%	1.8
Nutrition content	Protein	g	0
	Fat	g	0
	Carbohydrate	g	12.5
	Sodium	mg	575
	Potassium	mg	390
	Magnesium	mg	12
	Phosphorus	mg	31
pH	-	-	3.9
Osmolarity	-	mOsm	~270

Patient data were obtained from the electronic medical and anesthesia record systems. Extracted variables included demographic and clinical characteristics (age, sex, height, weight, BMI, medical history, ASA PS, preoperative diagnosis, dinner the night before surgery, use of preoperative laxatives, and use of diuretics such as sodium-glucose cotransporter 2 (SGLT2) inhibitors), ORS intake volume, anesthesia duration, surgery duration, type of anesthesia (inhalation anesthesia, inhalation plus epidural anesthesia, total IV anesthesia, total IV plus epidural anesthesia, or IV sedation under spontaneous breathing), anesthetic drug doses during induction (fentanyl and propofol), number of vasopressor administrations from anesthesia induction to surgery initiation, intraoperative infusion volume, urine output, and blood loss.

Patients were classified into two groups: those who received vasopressors after anesthesia induction (V group) and those who did not (N group). We examined whether differences in patient demographics and anesthesia-related factors influenced vasopressor administration. Our center’s anesthesia department criteria for vasopressor use were (i) a drop in systolic blood pressure after anesthesia induction of >20% from the preoperative baseline measured immediately after entering the operating room or (ii) mean arterial pressure below 60 mmHg. Phenylephrine hydrochloride (0.1 mg) and ephedrine hydrochloride (4-5 mg) were administered if the heart rate was below or above 60 bpm, respectively.

Primary and secondary outcomes

The primary outcome of this study was to clarify the dose-response relationship between ORS intake volume and vasopressor administration during the nonstimulus period from anesthesia induction to surgery initiation. Secondary outcomes included identifying patient- and anesthesia-related factors that influenced vasopressor administration.

Statistical analysis

For statistical analysis, the V and N groups were compared using unpaired t-tests or Pearson’s chi-squared tests. Factors associated with vasopressor administration immediately after anesthesia induction were identified using logistic regression with stepwise variable selection. To analyze the dose-response relationship between ORS intake and vasopressor administration, the relationship between ORS intake volume and vasopressor administration rate was visualized using a generalized additive model (GAM) with a binomial distribution and spline curves. A two-sided significance level of 5% was applied for all analyses. Statistical analyses were conducted using JMP version 10.0.2 (SAS Institute, Cary, NC, USA).

## Results

Based on the eligibility and exclusion criteria, 1,000 patients who underwent surgery between April 1, 2021, and December 27, 2021, were included in this study. Of these, 473 (47%) received vasopressors (0.1 mg of phenylephrine hydrochloride or 4-5 mg of ephedrine hydrochloride) between anesthesia induction and the start of surgery. Table 4 shows the average number of vasopressor administrations in each group.

**Table 4:** Status and frequency of vasopressor administration Data are presented as numbers or mean ± SDs.

Vasopressor	With medication (n, %)	No medication (n, %)	Number of administrations (times, mean ± SD)
	Group V	Group N	
Ephedrine hydrochloride	444 (44.4%)	556 (55.6%)	1.5 ± 0.7
Phenylephrine hydrochloride injection	154 (15.4%)	846 (84.6%)	1.5 ± 0.9

Regarding patient background factors (Table 5), no significant differences were observed between the two groups in sex, BMI, or the use of diuretics, SGLT2 inhibitors, or preoperative laxatives. However, compared to the N group, the V group had a higher average age (65.4 ± 12.2 vs. 58.7 ± 14.9 years, p < 0.0001), lower body weight (58.4 ± 11.4 vs. 61.0 ± 11.7 kg, p < 0.001), lower frequency of preoperative dinner consumption (424/473 vs. 506/527 cases, p < 0.0001), and higher severity according to the ASA PS I/II classification (124/349 vs. 203/324, p < 0.0001). 

**Table 5:** Background of study participants Data are presented as mean ± SDs. For continuous data, unpaired t-tests were used; for binary data, Pearson’s chi-squared tests were used. ASA PS, American Society of Anesthesiologists Physical Status; n.s., not significant; ORS, oral rehydration solution; SGLT2, sodium-glucose cotransporter 2; TIVA, total IV anesthesia

Variable	Vasopressor administration		p-Value
	Yes (Group V, n = 473)	No (Group N, n = 527)	
Sex (male/female) (%)	193 (40.8)/280 (59.2)	238 (45.2)/289 (54.8)	n.s.
Age (years)	65.4 ± 12.2	58.7 ± 14.9	<0.0001
Weight (kg)	58.4 ± 11.4	61.0 ± 11.7	<0.001
BMI (kg/m²)	22.9 ± 3.7	23.2 ± 3.5	n.s.
ASA PS status (I/II) (%)	124 (26.2)/349 (73.8)	203 (38.5)/324 (61.5)	<0.0001
Preoperative dinner (yes/no) (%)	424 (89.6)/49 (10.4)	506 (96.0)/21 (4.0)	<0.0001
Preoperative diagnosis	-	-	<0.0001
Urological cancer (%)	95 (20.1)	148 (28.1)	
Breast cancer (%)	67 (14.2)	140 (26.6)	
Respiratory cancer (%)	122 (25.8)	28 (5.3)	
Gynecological cancer (%)	54 (11.4)	68 (12.9)	
Hepatobiliary, pancreatic, digestive system cancer (%)	69 (14.6)	36 (6.8)	
Head and neck cancer (%)	38 (8.0)	63 (12.0)	
Bone and soft tissue cancer (%)	26 (5.5)	43 (8.2)	
Other (%)	2 (0.4)	1 (0.2)	
Diuretics (yes/no) (%)	2 (0.4)/471 (99.6)	3 (0.6)/524 (99.4)	n.s.
SGLT2 inhibitors (yes/no) (%)	8 (1.7)/465 (98.3)	10 (1.9)/517 (98.1)	n.s.
Anesthesia method	-	-	<0.0001
General anesthesia (inhalation) (%)	210 (44.4)	274 (52.0)	
General anesthesia (TIVA) (%)	109 (23.0)	115 (21.8)	
General anesthesia + epidural (%)	147 (31.1)	70 (13.3)	
IV anesthesia under spontaneous ventilation (%)	7 (1.5)	68 (12.9)	
Propofol 1% IV injection (mg/kg)	1.63 ± 0.42	1.82 ± 0.52	<0.0001
Fentanyl injection (0.1 mg/kg)	2.57 ± 1.19	2.34 ± 1.08	<0.01
Preoperative laxatives (yes/no) (%)	180 (38.1)/293 (61.9)	229 (43.5)/298 (56.5)	n.s.
Intraoperative fluid volume (mL/h)	569 ± 263	540 ± 260	n.s.
Intraoperative urine volume (mL/h)	81.1 ± 78.7	69.2 ± 83.0	<0.05
Intraoperative blood loss (mL/h)	41.0 ± 65.5	41.4 ± 88.1	n.s.
Operative duration (min)	187 ± 127	134 ± 122	<0.0001
ORS intake volume (mL)	996 ± 319	1055 ± 331	<0.05

Regarding preoperative cancer diagnoses, respiratory system cancer was the most common in the V group, whereas breast cancer was the most common in the N group. For anesthetic drug administration during induction, the V group received significantly lower doses of propofol (1.63 ± 0.42 vs. 1.82 ± 0.52 mg/kg, p < 0.0001) and fentanyl (2.57 ± 1.19 vs. 2.34 ± 1.08 μg/kg, p < 0.01) than the N group. Inhalation anesthesia was the most common method of maintenance in both groups; however, the V group had a higher proportion of patients receiving combined epidural anesthesia.

**Figure 1:**
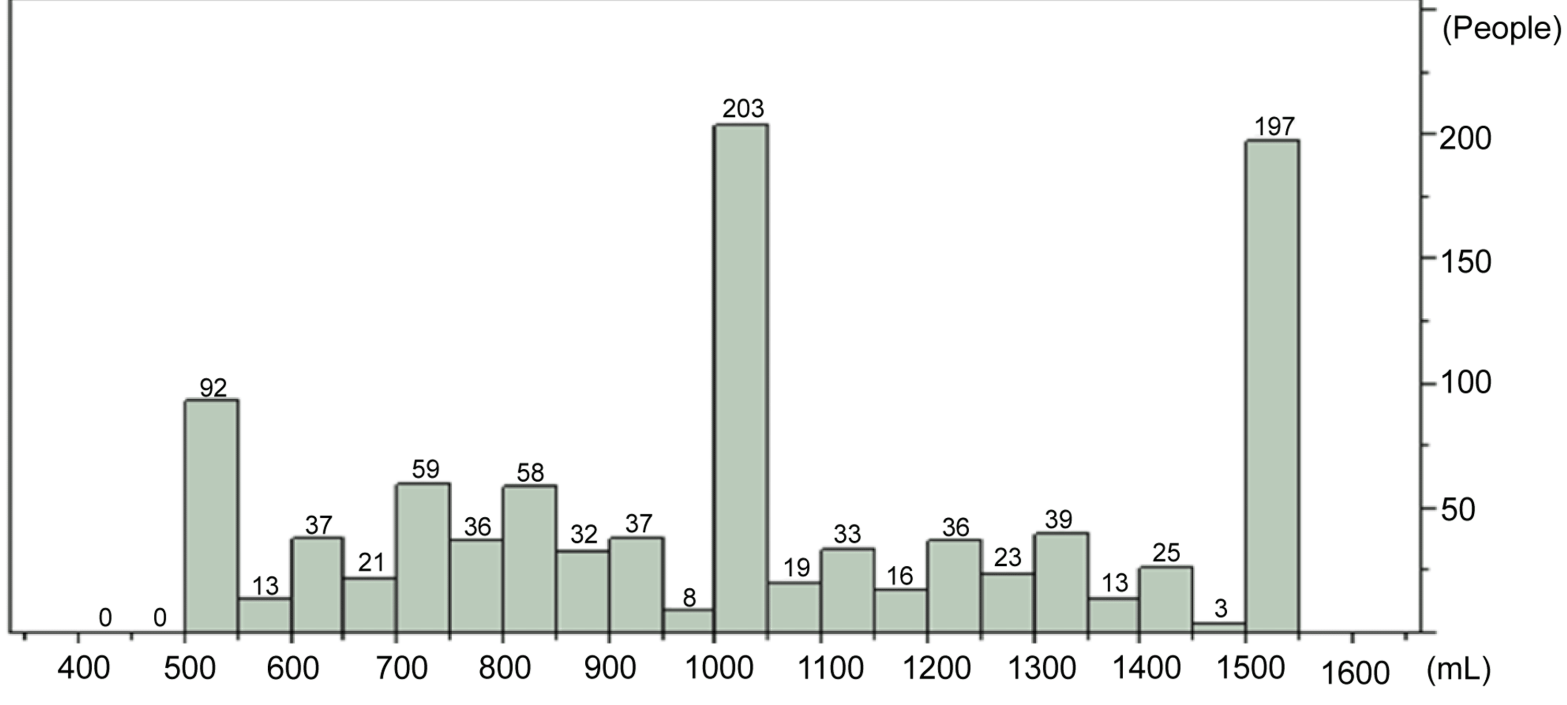
Distribution of ORS intake Among the 1,000 patients included in this study, 393 (39.3%) consumed 500-<1,000 mL of ORS, 410 (41.0%) consumed 1,000-<1,500 mL, and 197 (19.7%) consumed ≥1,500 mL. ORS, oral rehydration solution

Regarding intraoperative findings, no significant difference was observed in blood loss between the groups. However, the V group had significantly higher intraoperative urine output (569 ± 263 vs. 540 ± 260 mL, p < 0.05) and a longer surgical time (187 ± 127 vs. 134 ± 122 min, p < 0.0001). The distribution of ORS intake among patients was as follows: 500-999 mL (n = 393, 39%), 1,000-1,499 mL (n = 410, 41%), and ≥ 1,500 mL (n = 197, 20%) (Figure 1). 

The primary outcome of this study was to analyze the dose-response relationship between ORS intake and vasopressor administration rate during the nonstimulus period from anesthesia induction to surgery initiation. The results showed that ORS intake up to 800 mL increased the rate of vasopressor administration from 50% to 52%. However, intake above 800 mL reduced the vasopressor administration rate, reaching a minimum of 40% (p = 0.0024) (Table 6). Logistic regression analysis further revealed that when ORS intake exceeded 800 mL, each additional 100 mL was associated with a 0.96-fold reduction in the vasopressor administration rate (Table 6).

**Table 6:** Unadjusted logistic model The response variable was the presence or absence of vasopressor administration. AIC = 1654.75 AIC, Akaike information criterion; ORS, oral rehydration solution

Item	Population estimate	95% CI lower limit	95% CI upper limit	p-Value	OR	95% CI lower limit	95% CI upper limit
Intercept	-0.6152	-0.7346	-0.4958	<0.0001	-	-	-
ORS < 800 (/100)	0.028	-0.05	0.106	0.4815	1.03	0.95	1.11
ORS ≥ 800 (/100)	-0.0458	-0.0754	-0.0162	0.0024	0.96	0.93	0.98

For the secondary outcome, which examined patient background and anesthesia-related factors influencing vasopressor administration, multivariable analysis showed that ORS intake of ≥ 800 mL was significantly associated with a lower frequency of vasopressor administration. In contrast, epidural anesthesia, older age, and female sex were significantly associated with a higher frequency of vasopressor administration (Table 7). 

**Table 7:** Multivariate logistic regression analysis of factors related to vasopressor administration ORS, oral rehydration solution

Variable	Items	Logistic parameters			p-value	OR		
		Estimated value	95% lower limit	95% upper limit		OR	Lower limit	Upper limit
Intercept	-	-1.6412	-2.6522	-0.6302	0.0015	-	-	-
ORS < 800 (/100)	-	0.0338	-0.1378	0.2054	0.6995	1.034	0.871	1.228
ORS ≥ 800 (/100)	-	-0.073	-0.1361	-0.0099	0.0233	0.930	0.873	0.990
Preoperative diagnosis (control: gynecological cancer)	Other	-0.5802	-1.8028	0.6424	0.3523	0.560	0.165	1.901
	Hepatobiliary and pancreatic cancer	0.3267	-0.3329	0.9864	0.3316	1.386	0.717	2.681
	Respiratory system cancer	0.8583	-0.1132	1.8298	0.0833	2.359	0.893	6.233
	Bone and soft tissue cancer	-0.6989	-1.7023	0.3045	0.1722	0.497	0.182	1.356
	Head and neck cancer	-0.9038	-1.8526	0.0451	0.0619	0.405	0.157	1.046
	Breast cancer	-1.253	-2.1264	-0.3796	0.0049	0.286	0.119	0.684
	Urological cancer	0.2981	-0.3873	0.9835	0.394	1.347	0.679	2.674
Anesthesia method (control: general anesthesia + epidural anesthesia)	IV anesthesia under natural airway	-2.9132	-3.8568	-1.9696	<0.0001	0.054	0.021	0.140
	Total IV anesthesia	-0.6198	-1.1301	-0.1096	0.0173	0.538	0.323	0.896
	Total IV anesthesia (inhalation)	-0.6705	-1.1186	-0.2224	0.0034	0.511	0.327	0.801
Age	-	0.0406	0.0291	0.0522	<0.0001	1.041	1.029	1.054
Sex (control: male)	Female	0.5831	0.1672	0.9991	0.006	1.792	1.182	2.716
Preoperative laxative (control: none)	Yes	-0.6505	-1.3034	0.0024	0.0509	0.522	0.272	1.002

## Discussion

This study retrospectively examined the relationship between ORS intake and hemodynamic fluctuations during anesthesia induction in 1,000 patients undergoing elective surgery. The results confirmed the hypothesis that “appropriate preoperative ORS intake reduces circulatory suppression from anesthesia induction to the start of surgery.” Furthermore, it was demonstrated that consuming 800-1,500 mL of ORS before surgery could mitigate circulatory suppression.

Guidelines published by the American Society of Anesthesiologists [9], the European Society of Anaesthesiology [10], and the Japanese Society of Anesthesiologists [4] state that clear fluids can be safely consumed up to two hours before anesthesia induction, but they do not specify an exact intake volume. Based on this study’s findings, consuming at least 800 mL of ORS (a type of clear fluid) after the preoperative dinner may help prevent hypotensive events during anesthesia induction in adults. Regarding the upper limit of intake, this study was limited to a maximum of 1,500 mL due to financial constraints at our center, which prevented investigation of larger volumes. However, among patients who consumed 1,500 mL of ORS, the vasopressor administration rate was 39.1% (77/197 cases), indicating a 12.8% reduction in vasopressor use, representing a high relative risk reduction of 25% ((1 - (39.1/51.9)) × 100). Additionally, since vasopressor administration decreased 0.96-fold for every additional 100 mL of ORS intake, a higher intake volume could further reduce the risk. Based on these findings, it is recommended that adults consume at least 800 mL of ORS before surgery, with intake as close to 1,500 mL as possible between the preoperative dinner and two hours before anesthesia induction.

Traditionally, the Holliday-Segar formula has been used to calculate maintenance fluid requirements for preoperative fluid management [11]. According to this formula, the estimated maintenance fluid requirement is 100 mL per hour for an adult weighing 60 kg, which is close to the average body weight of the study participants. Since the body fluid maintenance period in this study lasted 12-18 hours, the estimated fluid requirement via maintenance IV infusion would have been 1,200-1,800 mL. However, most patients had consumed dinner, likely providing an additional 300-500 mL of fluid from food, which explains why the recommended ORS intake in this study was lower than the calculated fluid requirement.

Compared to daily life, the preoperative period involves extended fasting, and patients may also receive laxatives, increasing the risk of dehydration [5,6]. Consequently, the likelihood of hypotensive events during anesthesia induction, a key focus of this study, may also be heightened. Numerous previous studies have reported that intraoperative hypotensive events adversely affect postoperative morbidity and mortality [7,12-14]. In particular, for patients undergoing noncardiac surgery, intraoperative hypotension, defined either by a drop from baseline blood pressure or an absolute threshold, is associated with postoperative acute kidney injury and myocardial injury. While there is no universal definition for intraoperative hypotension, Südfeld et al. [15] defined early intraoperative hypotension as events occurring within one to 30 minutes after general anesthesia induction. In this study, although we did not set a strict timeframe, hypotensive events were defined as occurring immediately after anesthesia induction until the start of surgery, largely consistent with Südfeld’s definition.

According to Südfeld et al., factors contributing to hypotensive events after anesthesia induction include emergency surgery, higher ASA PS severity, neuraxial anesthesia, older age, and preinduction hypotension. Of these, preinduction hypotension is attributed to inadequate preoperative fluid loading, which leads to a fluid deficit [15]. This aligns with our findings that insufficient ORS intake and consequent body fluid insufficiency resulted in a higher frequency of hypotensive events. Furthermore, Szabó et al. demonstrated that preoperative fluid management with IV fluids can correct preoperative dehydration and prevent hypotensive events through appropriate fluid loading [16]. Using an ultrasound-based protocol, they evaluated inferior vena cava and lung ultrasound profiles as indicators of preoperative fluid status, allowing quantitative assessment. Given that preoperative dehydration due to insufficient fluid loading increases hypotensive events and negatively impacts postoperative morbidity and mortality, this study’s findings suggest that preoperative ORS intake of at least 800 mL could serve as a viable strategy for promoting postoperative recovery.

Our findings showed that the incidence of hypotensive events was significantly higher among patients with lower ORS intake, those receiving combined epidural anesthesia, older adults, female patients, patients with higher ASA PS severity, patients with lower body weight, and those who did not consume dinner the night before surgery. The higher incidence of hypotensive events in patients receiving epidural anesthesia, older patients, and those with higher ASA PS severity is consistent with the results of Südfeld et al. [15]. Epidural anesthesia suppresses sympathetic nervous activity, older patients have decreased cardiovascular and autonomic function, and higher ASA PS severity is associated with more cardiovascular comorbidities. In addition to these factors, preoperative dehydration further exacerbated the frequency of hypotensive events.

The finding that female patients experienced more hypotensive events differed from Südfeld et al. [15], who reported no significant impact of sex. We were unable to provide a clear explanation for this difference. Patients who did not eat dinner the night before surgery had a higher incidence of hypotensive events, likely due to reduced fluid intake from food. Additionally, the lower incidence of hypotensive events in patients undergoing breast cancer surgery may be attributed to the absence of epidural anesthesia.

Regarding medications administered during anesthesia induction, in the V group, propofol doses were lower, whereas fentanyl doses were higher, suggesting a potential influence on hemodynamics. According to Reich et al., propofol and high-dose fentanyl (>5.0 μg/kg) are risk factors for hypotensive events during anesthesia induction [17]. However, in this study, although fentanyl doses were higher in the V group, none of the patients received doses exceeding 5.0 μg/kg, suggesting similar effects on hypotensive events between groups. We did not investigate the concentration of volatile anesthetics immediately after induction, as Jor et al. concluded that the slow onset of volatile anesthetic effects makes their influence minimal [18].

**Figure 2:**
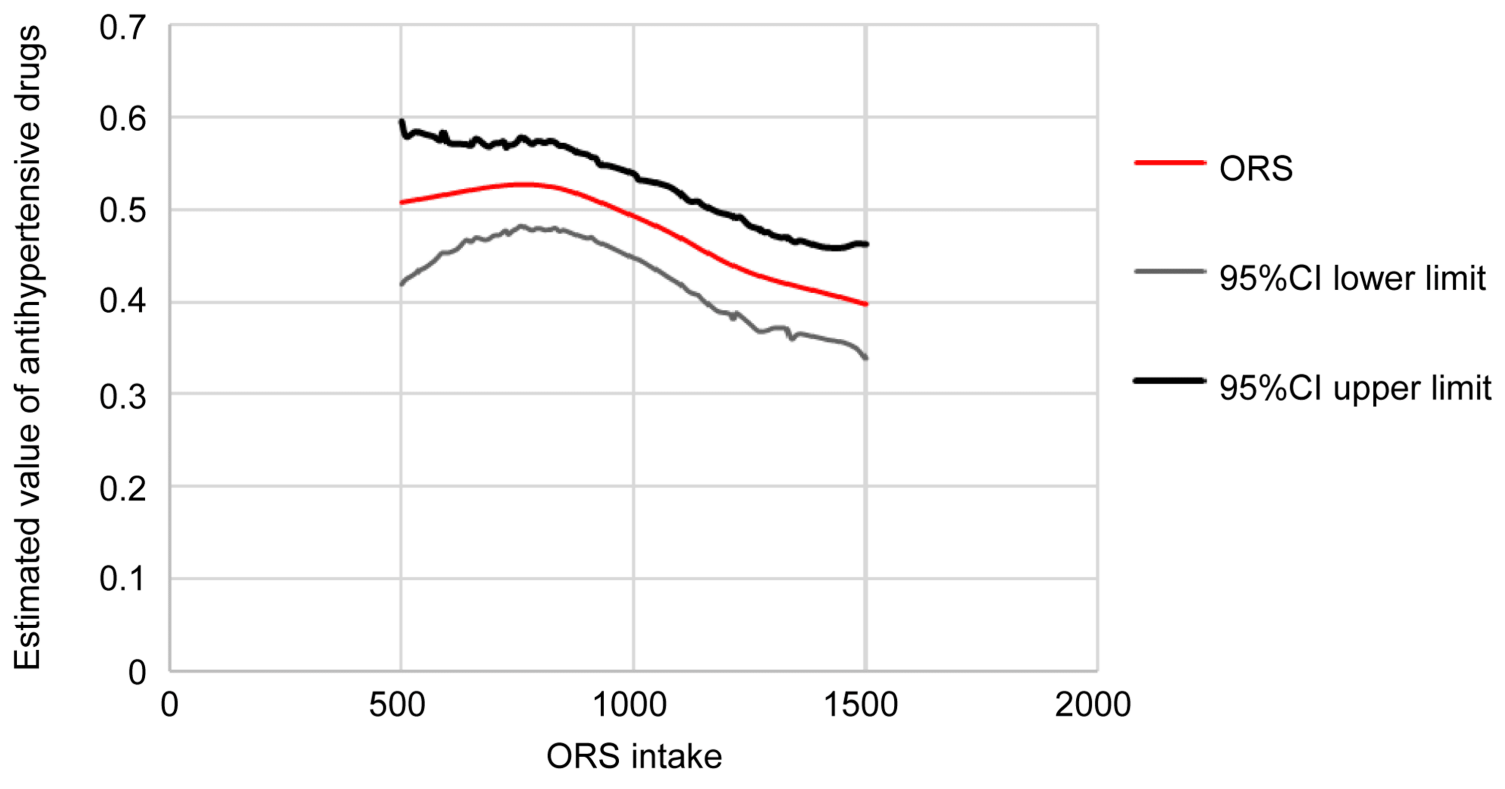
Relationship between ORS intake and vasopressor use - GAM and binomial regression This figure shows the relationship between ORS intake and the proportion of patients requiring vasopressors using a GAM with a binomial distribution and a spline curve (95% confidence interval obtained through 1,000 bootstrap simulations). The proportion of patients requiring vasopressors remained relatively stable (approximately 50-52%) for ORS intake between 500 and 800 mL. Beyond 800 mL, the proportion gradually declined, reaching 40% at 1,500 mL. GAM, generalized additive model; ORS, oral rehydration solution

In our study, we hypothesized that vasopressor administration depended on ORS intake volume. We plotted a dose-response curve to determine a threshold. The GAM model used identified relationships between predictors and responses across datasets without assuming a specific model [19]. Using spline curves, ORS intake was represented as a smoothed curve with 10 mL increments rather than a bar graph (Figure 2), without assuming linearity. This analysis identified approximately 800 mL of ORS as the threshold for vasopressor administration. Further multivariate logistic regression analyses supported these findings. Thus, the GAM approach was an appropriate method to visualize trends and derive the study’s conclusions.

Limitations

This study has four main limitations. First, as a retrospective study, we could not evaluate whether fluid correction using POORT was appropriately conducted. Therefore, we were unable to directly demonstrate the relationship between preoperative dehydration and hypotensive events. This relationship could have been assessed using quantitative ultrasound, as employed by Szabó et al. [16]. Nevertheless, the value of this study lies in its ability to assess the indirect impact of ORS intake on hypotensive events in a large cohort of 1,000 patients.

Second, the ORS volume provided in POORT was standardized at 1,500 mL without considering individual patient differences. At our center, a fixed ORS volume was used for each patient, as individual adjustments would complicate operations and increase the risk of error. Consequently, we could not propose individualized ORS intake recommendations. A prospective study is necessary to establish guidelines for ORS intake based on body weight.

Third, we did not examine cases in which ORS intake exceeded 1,500 mL. Due to institutional distribution policies and economic constraints, the maximum ORS volume provided was set at 1,500 mL. Based on our findings, higher ORS intake could potentially prevent hypotensive events in high-risk patients. A prospective study using methods described by Szabó et al. [16] would be necessary to evaluate the effects of ORS intake above 1,500 mL.

Fourth, reproducibility may be limited because vasopressor administration was used as a surrogate outcome, which involves subjective clinical judgment. Although the criteria for vasopressor administration were standardized, as described in the Methods section, anesthesiologists’ discretion cannot be fully excluded. Ideally, this assessment should have been conducted as an observational component within a prospective interventional study. However, as this study was designed to be exploratory and hypothesis-generating, the findings still possess academic value.

## Conclusions

An appropriate preoperative ORS intake has the potential to mitigate circulatory suppression during anesthesia induction. In this analysis of 1,000 patients undergoing elective surgery, an ORS intake volume of 800-1,500 mL was associated with lower vasopressor administration during anesthesia induction. The risk of hypotensive events was particularly high in patients receiving total IV anesthesia and combined epidural analgesia, older patients, underweight patients, those who did not consume dinner the night before surgery, and those with higher ASA PS severity.
